# SARS-CoV-2 infects human primary cytotrophoblasts mainly through a non-canonical entry route

**DOI:** 10.1093/molehr/gaag015

**Published:** 2026-02-26

**Authors:** Hélène Pinatel, Marie-Eve Brien, Mathilde Broquière, Marie-Pier Scott-Boyer, Arnaud Droit, Sylvie Girard, Géraldine Delbès, Laurent Chatel-Chaix, Cathy Vaillancourt

**Affiliations:** Centre Armand-Frappier Santé Biotechnologie, Institut national de la recherche scientifique, Laval, QC, Canada; CIUSSS-Nord-de-l’île-de-Montréal (Hôpital Sacré-Cœur), Montréal, QC, Canada; Sainte-Justine Hospital Research Center, Montréal, QC, Canada; Centre Armand-Frappier Santé Biotechnologie, Institut national de la recherche scientifique, Laval, QC, Canada; Département de médecine moléculaire, Université Laval, Québec, QC, Canada; Axe Endo-Nephro, Centre de recherche du CHU de Québec-Université Laval, Québec, QC, Canada; Département de médecine moléculaire, Université Laval, Québec, QC, Canada; Axe Endo-Nephro, Centre de recherche du CHU de Québec-Université Laval, Québec, QC, Canada; Sainte-Justine Hospital Research Center, Montréal, QC, Canada; Department of Obstetrics and Gynecology, Mayo Clinic, Rochester, MN, USA; Department of Immunology, Mayo Clinic, Rochester, MN, USA; Centre Armand-Frappier Santé Biotechnologie, Institut national de la recherche scientifique, Laval, QC, Canada; Centre Armand-Frappier Santé Biotechnologie, Institut national de la recherche scientifique, Laval, QC, Canada; Centre Armand-Frappier Santé Biotechnologie, Institut national de la recherche scientifique, Laval, QC, Canada; CIUSSS-Nord-de-l’île-de-Montréal (Hôpital Sacré-Cœur), Montréal, QC, Canada

**Keywords:** COVID-19, SARS-CoV-2, variants of concern, viral entry, cytotrophoblast, JEG-3 cells

## Abstract

Since the beginning of the COVID-19 pandemic, vulnerable populations such as pregnant persons have been at higher risk of severe symptoms and poor outcomes. Although reports of SARS-CoV-2 vertical transmission remain rare, several studies showed that maternal infection during pregnancy can induce histomorphological and inflammatory alterations in the placenta. However, the permissiveness of human trophoblasts to various variants of the virus remains poorly characterized. In this study, human primary villous cytotrophoblasts isolated from term placentas, along with trophoblastic cell lines BeWo, JEG-3, and HIPEC-65 were infected with the ancestral SARS-CoV-2 strain, which disseminated worldwide in early 2020. Permissiveness was assessed with quantitative RT-PCR, immunostaining of viral protein Nucleocapsid, and plaque assays. To investigate viral entry routes, cells were treated with Camostat mesylate (an inhibitor of the co-entry factor TMPRSS2) or chloroquine phosphate (an endosomal entry inhibitor) and viral fitness was assessed by plaque assays. Primary villous cytotrophoblasts and JEG-3 cells were also tested for infection with three pre-omicron SARS-CoV-2 variants of concern. Our results show that primary villous cytotrophoblasts are permissive to all tested SARS-CoV-2 strains *in vitro*. Infection with the ancestral SARS-CoV-2 strain relies mainly on a non-canonical endosomal entry pathway. Notably, JEG-3 cells represent an appropriate and convenient model for studying trophoblast infection by SARS-CoV-2, as they exhibit high permissiveness to the ancestral strain, and the SARS-CoV-2 entry pathway is similar to that in villous cytotrophoblasts. Overall, this study reveals that the cytotrophoblastic permissiveness to SARS-CoV-2 depends on the viral genetic nature and provides new insights into its entry route in human trophoblasts.

## Introduction

The 2020 major outbreak of severe acute respiratory syndrome coronavirus 2 (SARS-CoV-2) led to more than 777 million reported cases of coronavirus disease 2019 (COVID-19) and over 7 million deaths globally (WHO, 2025). Although the large-scale vaccination campaigns substantially reduced mortality (Tregoning *et al.*, 2021; WHO, 2025), SARS-CoV-2 continues to spread and evolve, giving rise to new variants. Several variants of concern (VOCs) have sequentially become predominant, and as of November 2025, variants such as XFG and NB.1.8.1 remain widely detected, and typically cause symptoms similar to those of seasonal influenza (CDC, 2024; WHO, 2025).

SARS-CoV-2 variants are characterized by mutations acquired into their genome, especially within the gene encoding the Spike (S) virion glycoprotein, which mediates entry of the viral particle (Harvey *et al.*, 2021). Two main entry routes have been described: a canonical pathway and a pH-dependent endosomal pathway. In the canonical route, the subunit S1 of Spike binds to the angiotensin-converting enzyme 2 (ACE2) receptor at the plasma membrane, followed by cleavage of subunit S2 by Transmembrane protease serine 2 (TMPRSS2), which induces the fusion between the viral envelope and the cellular plasma membrane (Hoffmann *et al.*, 2020; Walls *et al.*, 2020). In the non-canonical endosomal pathway, virus-bound to ACE2 is endocytosed and at low endosomal pH, the S2 domain of Spike is cleaved by the protease Cathepsin L (CTSL), allowing the subsequent fusion of the viral envelope with the endosomal membrane (Koch *et al.*, 2021). Variant-specific mutations in S protein can influence the cellular tropism of SARS-CoV-2 as well as the nature of the pathways used for its entry (Dubey *et al.*, 2021).

During the pandemic, pregnant persons did represent a vulnerable population with an elevated risk of severe COVID-19 outcomes (Allotey *et al.*, 2020; Ellington *et al.*, 2020). Despite some clinical case reports suggesting an *in utero* infection of the fetus associated with poor pregnancy outcomes (Correia *et al.*, 2020; Vivanti *et al.*, 2020; Morales *et al.*, 2021), extensive studies showed that vertical transmission was scarce (Raschetti *et al.*, 2020; Kotlyar *et al.*, 2021). However, the effects of maternal SARS-CoV-2 infection on the placental structure and function, despite their importance for fetal development, have been less characterized. Clinical studies reported histomorphological abnormalities in placentas from SARS-CoV-2-infected persons, such as fetal and maternal vascular malperfusion, chronic histiocytic intervillositis, massive fibrin deposits, and trophoblast necrosis (Brien *et al.*, 2021; Schwartz *et al.*, 2021; Wong *et al.*, 2021; Argueta *et al.*, 2022).

Experimental studies using *in vitro* and *ex vivo* models have explored whether SARS-CoV-2 can infect trophoblastic cells at various gestational stages. Organotypic precision-cut slices, villous explants, and primary trophoblasts cultured from term placentas have shown that SARS-CoV-2 can infect trophoblastic compartments, though reported permissiveness of syncytiotrophoblasts (STBs) and cytotrophoblasts (CTBs) varies between studies (Fahmi *et al.*, 2021; Lu-Culligan *et al.*, 2021; Argueta *et al.*, 2022). Investigations on innovative models of early pregnancy including human embryonic stem cell-derived trophoblasts and induced trophoblast stem cells from first-trimester placental tissue, indicated infection of STB but did yield diverging results regarding CTBs and extravillous trophoblasts (EVTs) (Zhou *et al.*, 2021; Chen *et al.*, 2023; Kallol *et al.*, 2023). Additional research is therefore needed to clarify the susceptibility of specific trophoblastic cell types across gestation and to determine how infection may impair placental development and function that could contribute to adverse pregnancy outcomes depending on the timing of infection and on the VOC. Moreover, most existing studies focused on the expression of the canonical entry factors (ACE2/TMPRSS2) in trophoblasts, but the precise entry mechanisms used by SARS-CoV-2 in these cells, as well as potential differences between the different Spike-diverging variants remain unknown. Furthermore, the availability of convenient, cost-effective, and reliable trophoblastic cellular models would greatly facilitate mechanistic studies of trophoblastic infection.

In this study, we compared the permissiveness of primary trophoblasts to multiple SARS-CoV-2 strains, including VOCs, and characterized the viral entry pathways involved using two commonly used trophoblastic *in vitro* models. We found that primary villous CTBs (vCTBs) isolated from placentas delivered at term from non-complicated pregnancies are permissive to the ancestral SARS-CoV-2 (PreVOC) strain that spread in early 2020, whereas viral permissiveness appeared decreased with the tested VOCs (Alpha, Beta, and Delta). We further demonstrate that PreVOC predominantly uses the endosomal route for entry into vCTBs, and that inter-individual variability among donors correlates with differential expression of viral entry factors. Among several trophoblastic cell lines tested, we identified the choriocarcinoma JEG-3 cells as an appropriate cost-effective model for trophoblastic SARS-CoV-2 infection. Similar to primary vCTBs, JEG-3 cell infection by SARS-CoV-2 predominantly relies on the endosomal entry pathway. Moreover, JEG-3 cells exhibit reduced permissiveness to Alpha and Delta variants compared to PreVOC.

## Materials and methods

### Placental tissue samples

Placental villous biopsies were collected from individuals who delivered between March 2020 and July 2021, as part of a cohort at CHU Sainte-Justine (Montreal, QC, Canada) (IRB number: MP-21-2019-1966). Written informed consent was obtained from all participants. This cohort has been previously described (Brien *et al.*, 2021, 2023). For the RNA sequencing analysis, placental biopsies from 26 SARS-CoV-2-negative donors were included. Biopsies were obtained rapidly after delivery and either snap-frozen or incubated in RNAlater (No. R0901, Sigma-Aldrich, Oakville, ON, Canada) for 24 h at 4°C before long-term storage at −80°C until further processing. For frozen samples, ∼20 mg of tissue was ground into powder on dry ice and subsequently homogenized in lysis buffer using the QIAshredder kit (No. 79656, QIAGEN, Toronto, ON, Canada). For RNAlater-preserved samples, small pieces of tissue were cut on dry ice and thawed in the RLT lysis buffer of the RNeasy Mini kit (No. 74106, QIAGEN) supplemented with 1% β-mercaptoethanol, in prefilled tubes containing garnet shards and a zirconium 6-mm bead (No. D1033-30G, Benchmark Scientific, Sayreville, NJ, USA). Mechanical lysis was performed using a BeadBug homogenizer (No. D1030, Benchmark Scientific), followed by additional homogenization using the QIAshredder columns (No. 79656, QIAGEN). RNA extraction was then performed as described below. Eight RNAlater-preserved biopsies were used for quantitative RT-PCR (RT-qPCR) analysis, including samples from two donors affected by intrauterine growth restriction and one by preeclampsia.

### Isolation of human primary vCTBs

All experiments with primary cells were performed according to the Declaration of Helsinki. All placentas were obtained following approval from the local ethics committee (Hôpital du Sacré-Cœur-de-Montréal (HSCM): 2019-1654 and 2021-2143; CHU Sainte-Justine (CHUSJ): 2015-771; Institut national de la recherche scientifique (INRS): 21-612) following patients’ written consent. Placental tissues were obtained from uncomplicated pregnancies with term delivery (>37 weeks of amenorrhea), within an hour after delivery by Cesarean Section. There was no available additional information on previous infection with SARS-CoV-2.

The vCTBs were isolated using sequential trypsin–DNase digestions and a subsequent Percoll gradient purification based on an adapted protocol from Kliman *et al.* (1986). This protocol has been previously described extensively in Sagrillo-Fagundes *et al.* (2016) and only minor modifications were made as follows: liquid trypsin was used for a more convenient dosing and a two-layer Percoll gradient was performed. Cells were frozen right after isolation and kept in liquid nitrogen until needed for experiments.

vCTBs do not express type I HLA, therefore they were purified by negative selection using anti-HLA-ABC antibodies (No. 14-9983-82, Thermo Fisher Scientific, Burlington, ON, Canada) and secondary antibodies coupled to microbeads (No. 130-048-4, Miltenyi Biotec, Bergisch Gladbach, Germany). Purified vCTBs were then cultured in Dulbecco’s Modified Eagle Medium (DMEM) High-Glucose (No. 10564011, Thermo Fisher Scientific), supplemented with 1% penicillin–streptomycin (No. 15140122, Thermo Fisher Scientific) and 10% fetal bovine serum (FBS) (No. 35-010-CV, Corning, New York, NY, USA). Cells were cultured in a humidified atmosphere containing 5% carbon dioxide (CO_2_) at 37°C and the medium was renewed 4 h after seeding to remove unattached cells, and every 24 h onward, vCTBs were seeded in 24-well plates (5×10^5^ cells/well in 500 µl) for viral replication evaluation and onto glass coverslips for microscopy analyses. For cytotoxicity assays, vCTBs were seeded in 96-well plates (1.25×10^5^ cells/well in 100 µl).

### Cell lines culture

BeWo (CCL-98 clone) and JEG-3 cell lines were obtained from the American Type Culture Collection (ATCC, USA) and cultured in DMEM/Ham’s F-12 (No. 319-085-CL, Wisent Inc., Saint-Jean-Baptiste, QC, Canada) supplemented with 10% FBS (No. 12484-028, Thermo Fisher Scientific) and in Essential Minimum Eagle Medium (EMEM) (No. 320-005-CL, Wisent Inc.) supplemented with 1 mM sodium pyruvate (No. 11360-070, Thermo Fisher Scientific), 10 mM HEPES (No. 15630-080, Thermo Fisher Scientific), and 10% FBS, respectively. HIPEC-65 cells were obtained from Thierry Fournier’s laboratory (University Paris Descartes, Paris, France) and were maintained in DMEM/Ham’s F-12 supplemented with 5% FBS. VeroE6 cells (CRL-1586, ATCC) and Huh7.5 cells (a gift from Patrick Labonté, INRS, Laval, Canada) were cultured in DMEM (No. 11965-092, Thermo Fisher Scientific) supplemented with 1% non-essential amino acids (No. 11140-050, Thermo Fisher Scientific), 1% penicillin–streptomycin (No. 15140-122, Thermo Fisher Scientific), and 10% FBS (No. 098150, Wisent Inc.).

Cells were cultured in a humidified atmosphere containing 5% CO_2_ at 37°C and passaged every 2–3 days (about 90% confluence) using TrypLE Express, a cell dissociation buffer (No. 12604-021, Thermo Fisher Scientific). For infection studies, cells were seeded in 6-well plates in 2 ml (BeWo cells: 1.5×10^5^ cells/well; JEG-3 cells: 2×10^5^ cells/well; HIPEC-65 cells: 1×10^5^ cells/well; Huh7.5 cells: 3×10^5^ cells/well) for RNA extraction and supernatant collection, and in 24-well plates onto glass coverslips for immunofluorescence (2×10^4^ cells/well in 500 µl). For cytotoxicity assay, cells were seeded in 96-well plates in 100 µl (Huh7.5 cells: 1×10^4^ cells/well; JEG-3 cells: 7×10^3^ cells/well).

### Viruses

SARS-CoV-2 variants (Table 1) were provided by the Public Health Laboratory of Quebec (LSPQ, Canada). Virus stocks were produced by amplification in VeroE6 cells following infection with a multiplicity of infection (MOI, i.e. the number of infectious particles per cell) of 0.1. Supernatants containing viral particles were filtered at 0.45 µm, aliquoted, and stored at −80°C. Infectious titers were determined by plaque assays.

**Table 1.** SARS-CoV-2 strains used in this study.

Name	Variant	Strain
PreVOC (March 2020)	B.1 (D614G)	Canada/QC-L00214517/2020
Alpha	B.1.1.7	Canada/QC-L00329228/2021
Beta	B.1.351	Canada/QC-L00327509/2021
Delta	B.1.617.2	Canada/QC-L00414346/2021

PreVOC, ancestral SARS-CoV-2 strain.

### SARS-CoV-2 infection

Infection of primary vCTBs and cell lines was done in the containment level 3 facility of INRS with the approval of the institutional biosafety committee (certificate ID: 2021-01). Cells were exposed to SARS-CoV-2 at an MOI of 1 for 2–3 h. The virus inoculum was then removed and replaced by fresh medium. Over the course of infection, the supernatants and cell lysates were collected at 24, 48, and 72 h post-infection (hpi) and stored at −80°C until further analysis. For immunofluorescence analysis, cells were fixed in 4% paraformaldehyde (in phosphate buffer saline (PBS)) for 30 min, washed twice with PBS, and stored in PBS at 4°C for downstream immunostaining.

### Drug tests

As controls for viral replication, trophoblastic cell lines and primary vCTBs were exposed to 2 µM of antiviral drugs, dissolved in dimethyl sulfoxide (DMSO) after removal of the virus inoculum. Two FDA-approved drugs were used: Nirmatrelvir (NIR, also named PF-07321332; No. HY-138687, MedChemExpress, Monmouth Junction, NJ, USA) and Remdesivir (RMD), a pro-drug also called GS-5734 (No. DT-0049, Key Organics, Camelford, UK). Although both inhibit SARS-CoV-2, they act through distinct mechanisms. NIR is an inhibitor of the viral main protease (NSP5) and is clinically administered in combination with Ritonavir, an HIV-1 protease and cytochrome P450 3A inhibitor that increases NIR bioavailability (Owen *et al.*, 2021; Santé-Canada, 2022; Saravolatz *et al.*, 2023). RMD, initially developed for Ebola virus, is metabolized intracellularly into an adenosine analogue that inhibits the viral RNA-dependent RNA polymerase (RdRp) including SARS-CoV-2 (Warren *et al.*, 2016; Wang *et al.*, 2020).

To test viral entry routes, the vCTBS, Huh7.5, and JEG-3 cells were exposed for 1 h to 20 µM of Camostat mesylate (No. 16018, Cayman Chemical, Ann Arbor, MI, USA), chloroquine phosphate (No. 14194, Cayman Chemical), or their respective vehicle (DMSO or PBS). This concentration (20 µM) was chosen to ensure a complete inhibition of the canonical TMPRSS2-dependent entry pathway with Camostat mesylate treatment, and of the endosomal acidification-dependent entry route with chloroquine phosphate treatment. Cells were then exposed to the virus at an MOI of 1 for 2–3 h and to the drugs. Then, the media was replaced by fresh medium containing drugs. At 24, 48, and/or 72 hpi, the virus-containing supernatants were collected, and cells were lysed directly zin the plate in 350 µl of RLT buffer of the RNeasy Mini kit (No. 74106, QIAGEN) supplemented with 1% β-mercaptoethanol. Samples were stored at −80°C until further processing. Control cells were exposed to a final concentration of 0.1% of the vehicle (DMSO or PBS depending on the tested drugs).

### Drug-treated cell viability assays

To mimic the experimental design of the viral entry test, cells were exposed for 1 h to Camostat, chloroquine or the vehicle (pre-treatment). Then, the drug-containing media were renewed for another 2–3 h to simulate the infection step. Subsequently, the media and drugs were renewed one last time. Cells were also exposed to the antiviral drugs (NIR or RMD) or their vehicle at one time point. To assess cell viability, the CellTiter-Glo Luminescent Cell Viability Assay (No. G7571, Promega, Madison, WI, USA) was used according to the manufacturer’s instructions. The luminescence was measured with a Spark multimode microplate reader (Tecan, Morrisville, NC, USA).

### RNA extraction

Cells lysed in the supplemented RLT buffer (see above) were subjected to total RNA extraction with the RNeasy Mini kit (No. 74106, QIAGEN) according to the manufacturer’s instructions. RNA purity (OD_260/280_ ratio ≥ 1.8) and concentration (ng/μl) were determined using a NanoDrop instrument (Nanodrop Lite, Thermo Fisher Scientific).

### RT-qPCR

For measuring host gene expression, reverse transcription was performed using the iScript cDNA Synthesis Kit (No. 1708891, Bio-Rad, Mississauga, ON, Canada) with 0.5 µg of total RNA. cDNA was amplified using SsoAdvanced Universal SYBR Green Supermix (No. 1725274, Bio-Rad) with a CFX96 Real-Time PCR Detection System (BioRad). Primer pairs targeting the genes of interest *ACE2*, *TMPRSS2*, Neuropilin-1 (*NRP1)*, Scavenger receptor class B type 1 (*SCARB1)*, *CTSL*, and reference genes Tyrosine 3-Monooxygenase/Tryptophan 5-Monooxygenase Activation Protein Zeta (*YWHAZ*), and Succinate Dehydrogenase Complex Flavoprotein Subunit A (*SDHA*) are listed in Table 2 along with their respective annealing temperatures during PCR. cDNAs were diluted at 1:20 in water. PCR product sizes were verified by electrophoresis on a 2% agarose gel containing 0.01% of SYBR Safe (No. S33102, Thermo Fisher Scientific) and visualized under UV light.

**Table 2.** Primer sequences and optimal annealing temperatures (Ta) used for qPCR.

	Forward primer (5′–3′)	Reverse primer (5′–3′)	Ta
*ACE2*	AAATCCATTGGTCTTCTGTCACCC	ATCTCCCACCACTTTTTCATCCAC	58°C
*TMPRSS2*	AGACCAGGAGTGTACGGGAA	GCAAAACCAGCCCCATTGTT	58°C
*NRP1*	CCCTCACATTGGGCGTTACT	TATCGCGCTGTCGGTGTAAA	54°C
*SCARB1*	CTTGGCATTCACCACCCTCG	GTGAAGAGCCCAGAGTCGGA	58°C
*CTSL*	CGGCTTTGTGGACATCCCTA	AGGAAGGACTCATGACCTGC	58°C
*YWHAZ*	GGCAACCTAAGAACAAATG	CATGTTAGGCAAGTATCAAA	58°C
*SDHA*	GTTTGTTCAGTTCCACCCTACA	AAACCTTTCGCCTTGACTGTTA	54°C

For viral RNA detection, we used a TaqMan-based assay using probe labeled with 5′-6-carboxyfluorescein (FAM) fluorescent dye and 3′ Blackberry Quencher (BBQ) specific to the SARS-CoV-2 RNA coding for envelope (E) protein (Table 3) (Corman *et al.*, 2020). One-step RT-qPCR was performed with 5 µl of total RNA from primary vCTBs or 150 ng of total RNA for cell lines using the TaqPath 1-Step Multiplex Master Mix No ROX (No. A28523, Thermo Fisher Scientific) according to the manufacturer’s instructions. Relative quantification of viral mRNA was normalized to the uninfected condition.

**Table 3.** Primers and fluorescent probe sequences for SARS-CoV-2 detection by RT-qPCR.

	Identifying name	Sequence (5′–3′)
Forward primer	E_Sarbeco_F1	ACAGGTACGTTAATAGTTAATAGCGT
Reverse primer	E_Sarbeco_R1	ATATTGCAGCAGTACGCACACA
Probe	E_Sarbeco_P1	FAM-ACACTAGCCATCCTTACTGCGCTTCG-BBQ

### Plaque assays

VeroE6 cells were seeded in 24-well plates (2×10^5^ cells/well in 500 µl) and transferred to the INRS containment level 3 facility after 24 h. Cells were infected in triplicates with 200 µl virus samples that had been serially diluted (10^1^ to 10^4^ or 10^3^ to 10^6^) in complete medium. Two hours post-infection, the inoculum was removed and replaced with serum-free minimum essential medium (No. 11095-080, Thermo Fisher Scientific) containing 0.8% of carboxymethylcellulose (No. 21902, Sigma-Aldrich). Three days later, cells were fixed for 1 h with 5% formaldehyde (No. BP531500, Thermo Fisher Scientific), allowing complete inactivation of the infectious samples. The plates were then transferred outside the facility. Following several washes with tap water, cells were stained with 1% crystal violet/10% ethanol for 15 min and gently washed under tap water. Plaques were counted manually to calculate titers of infectious virus in plaque-forming units (PFU) per ml.

### Immunofluorescence

vCTBs were fixed for 30 min with 4% paraformaldehyde (in PBS). Following two washes with PBS, samples were kept at 4°C protected from light until the labeling. Cells were permeabilized with 0.2% Triton X-100 in PBS for 15 min, at room temperature (RT) and then blocked with PBS supplemented with 10% goat serum, 5% bovine serum albumin (BSA), and 0.05% sodium azide for 1 h. vCTBs were incubated for 2 h at RT protected from light with mouse anti-Nucleocapsid (No. 40143-MM05, Sino Biologicals, Paoli, PA, USA; diluted 1:300) primary antibodies in PBS containing 5% BSA and 0.05% sodium azide. Coverslips were washed 3 times with PBS before a 1-h incubation, protected from light, with donkey anti-mouse IgG Alexa Fluor 488-conjugated secondary antibodies (No. A21202, Thermo Fisher Scientific; diluted 1:1000). Samples were washed 3 times in PBS for 15 min in between each wash and were incubated with 1 µg/ml DAPI (No. D1306, Thermo Fisher Scientific) in PBS for 10 min. Lastly, cells were washed 3 times with PBS and once with water before being mounted on slides with Fluoromont G (No. 0100-01, SouthernBiotech, Birmingham, AL, USA). Imaging was performed using a 63× objective on an LSM980 confocal microscope (Zeiss, Toronto, ON, Canada) located at the Confocal Microscopy Core Facility of the INRS-Centre Armand-Frappier Santé Biotechnologie. Images were processed with the Fiji software (Schindelin *et al.*, 2012).

### mRNA sequencing

Human, mouse, and rat rRNA-depleted libraries were generated from 125 ng of total RNA and sequenced with an Illumina NovaSeq platform using paired-end 100 bp reads at Genome Quebec (Montreal, Quebec, Canada). Reads were trimmed using fastp v0.23.2 (Chen *et al.*, 2018). Quality check was assessed before and after trimming FastQC v0.11.9 (Andrews, 2010) and summarized with MultiQC v1.12 (Ewels *et al.*, 2016). Transcript identification and quantification were performed using Kallisto v0.48.0 (Bray *et al.*, 2016) against the Homo sapiens GRCh38 transcriptome (Ensembl release 110: Ensembl110_110.protein_coding). LogCPM values were calculated using the edgeR package. All downstream R analysis were conducted in R v4.3.1 (R Core Team, 2014).

### Comparative Transcriptomic Placental Model Atlas

The Comparative Transcriptomic Placental Model Atlas (CTPMA) is a recently reported tool enabling transcriptomic comparison across multiple trophoblastic models (Lapehn *et al.*, 2025). This atlas integrates RNA sequencing data from 22 datasets, comparing 1083 placental tissues, 8 villous explants, 36 primary trophoblastic cells, 14 trophoblast stem cells, and cell lines including 23 HTR8/SVneo, 16 JEG-3, 13 JAR, and 43 BeWo generated by multiple independent studies. We used the ‘Explore gene level expression’ tool to visualize the expression distribution of each gene of interest across placental models, and the ‘Explore user-defined list of gene’ tool to generate expression heatmap for comparative analysis.

### Statistical analysis

GraphPad Prism 10 software (version 10.5.0 for Windows, GraphPad Software, Boston, MA, USA) was used to perform statistical analysis. Differences between the groups were determined by unpaired, two-tailed Mann–Whitney test. A *P*-value < 0.05 was considered statistically significant.

## Results

### Primary villous trophoblasts are permissive to SARS-CoV-2

**Figure 1.**
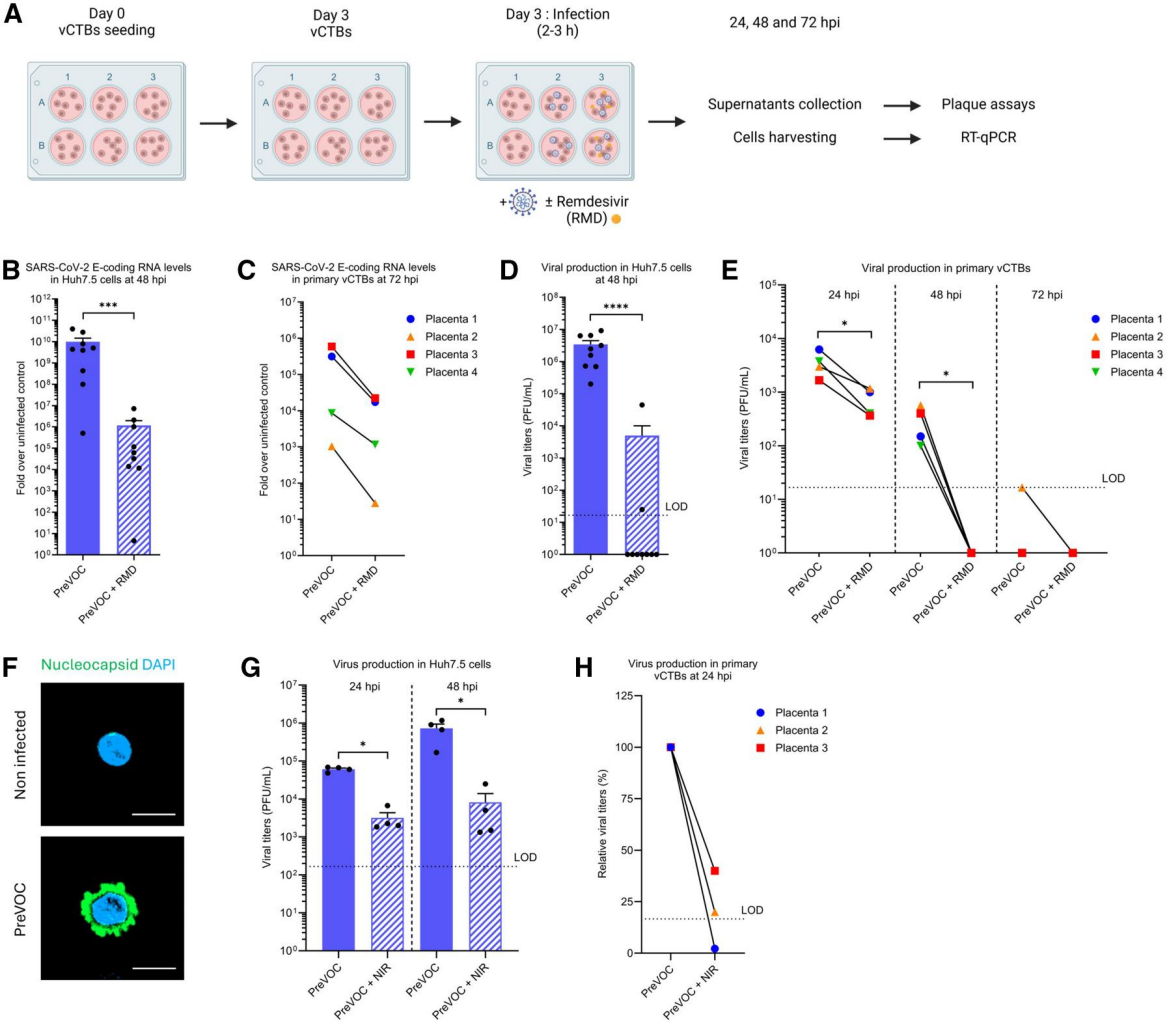
**Human primary cytotrophoblasts are permissive to SARS-CoV-2 PreVOC strain *in vitro***. (**A**) Timeline of primary trophoblast cells culture and infection: primary vCTBs were cultured for 72 h, then the cells were infected at an MOI of 1 with the ancestral SARS-CoV-2 (PreVOC) strain for 2-3 h and the media was renewed without or with 2 µM of Remdesivir (RMD). RMD treatment was used as a positive control for viral replication. Supernatants were collected and cells were harvested at 24, 48, and 72 hpi. Created in BioRender. Vaillancourt, C. (2026) https://BioRender.com/hi4f63i. (**B**, **D**) Huh7.5 cells (n = 9) were infected with SARS-CoV-2 PreVOC strain at an MOI of 0.1, as a positive control of SARS-CoV-2 infection. Viral replication of SARS-CoV-2 was evaluated by diagnostic RT-qPCR detecting E-coding RNA levels in Huh7.5 cells (n = 9) at 48 hpi (**B**) and (**C**) in vCTBs isolated from placentas delivered at term without complication, at 72 hpi. Each symbol represents a placenta from an individual donor (n = 4). (**D**, **E**) Infectious viral titers were determined using plaque assay at 48 hpi for Huh7.5 cells (**D**) or every 24 h for the vCTBs (**E**). When no viral titer was detected, a value of 1 was arbitrarily assigned. (**F**) Infected cells were detected by immunostaining of the Nucleocapsid SARS-CoV-2 protein in green and cell nuclei were counterstained with DAPI (blue). Cells were observed with confocal microscopy at 63× objective. Scale bar 10 µm. (**G**, **H**) Infected cells were treated with 2 µM of Nirmatrelvir (NIR) to confirm active viral replication within Huh7.5 cells (**G**) and primary vCTBs (**H**). Plaque assays were performed to evaluate the viral production. The black dashed line represents the limit of detection (LOD) of the plaque assay. Data are shown as mean ± SEM or as individual symbol for vCTBs (*P < 0.5, ***P < 0.001, ****P < 0.0001; Mann–Whitney test). hpi, hours post-infection; MOI, multiplicity of infection; PFU, Plaque-forming unit; vCTBs, villous cytotrophoblasts.

To study the susceptibility of primary vCTBs to SARS-CoV-2, cells were infected with the PreVOC strain at an MOI of 1 for 2–3 h and harvested at 24, 48, and 72 hpi (Fig. 1A). SARS-CoV-2-permissive Huh7.5 hepatocarcinoma cells (Dittmar *et al.*, 2021) infected with an MOI of 0.1, served as a positive control. To ensure that detected viral signals reflected productive replication rather than residual inoculum, infected cells were treated in parallel with RMD, a viral polymerase inhibitor approved for COVID-19 treatment (Agarwal *et al.*, 2020). Viral replication was first quantified by one-step TaqMan RT-qPCR targeting SARS-CoV-2 subgenomic RNA coding for the E viral protein, used for diagnosis of SARS-CoV-2 infection. As expected, Huh7.5 cells displayed high levels of viral RNA at 48 hpi, which were reduced by more than 99% upon RMD treatment (Fig. 1B).

In primary vCTBs, viral RNA was also readily detected at 72 hpi, yet at lower levels than in Huh7.5 cells. Most importantly, this amount was reduced upon RMD treatment by around 95% (Fig. 1C), demonstrating active viral RNA replication of the PreVOC strain in vCTBs. Plaque assays at different time points post-infection further validated the production and release of new infectious viral particles in both Huh7.5 cells (Fig. 1D) and vCTBs (Fig. 1E). In primary vCTBs at 24 and 48 hpi, extracellular infectious viral particles were detected in the culture medium, and their abundance was significantly decreased upon RMD treatment (Fig. 1E), demonstrating that they result from an active viral replication and not from the primary virus inoculum used for infection. This result highlights that the SARS-CoV-2 PreVOC strain can complete a full replication cycle in trophoblastic cells *in vitro*. Viral yield in vCTBs was significantly lower than in Huh7.5 cells and decreased over time. It is noteworthy that, although viral particles were specifically and readily detected, the efficiency of virus production in primary trophoblasts was low compared to that from the infected Huh7.5 (Fig. 1D). Moreover, in vCTBs, the highest virus yields were observed at 24 hpi and titers decreased over time, being under the limit of detection at 72 hpi. This suggests that SARS-CoV-2 replication is not highly efficient and sustainable in trophoblast cells. Therefore, the best time point to study trophoblast infection *in vitro*, that is the peak of replication, is 24 hpi. To visualize infection, confocal microscopy was performed using anti-Nucleocapsid (N) antibodies and DAPI. vCTBs showed strong N staining at 24 hpi (Fig. 1F), consistent with active viral RNA synthesis and production of N subgenomic transcripts. As an additional replication control, cells were treated with another FDA-approved anti-SARS-CoV-2 drug Nirmatrelvir (NIR), an inhibitor of the main SARS-CoV-2 protease (Owen *et al.*, 2021). NIR significantly reduced infectious virus production in both Huh7.5 cells (Fig. 1G) and vCTBs (Fig. 1H), further confirming productive SARS-CoV-2 replication in primary trophoblasts. These results provide strong evidence of a productive SARS-CoV-2 life cycle in primary human vCTBs.

### SARS-CoV-2 permissiveness diverges among trophoblastic cell lines

Cell lines are widely used in the field of human placental research. They often constitute a cost- and time-effective alternative, and their various characteristics allow them to mimic trophoblastic cell subtypes.

**Figure 2.**
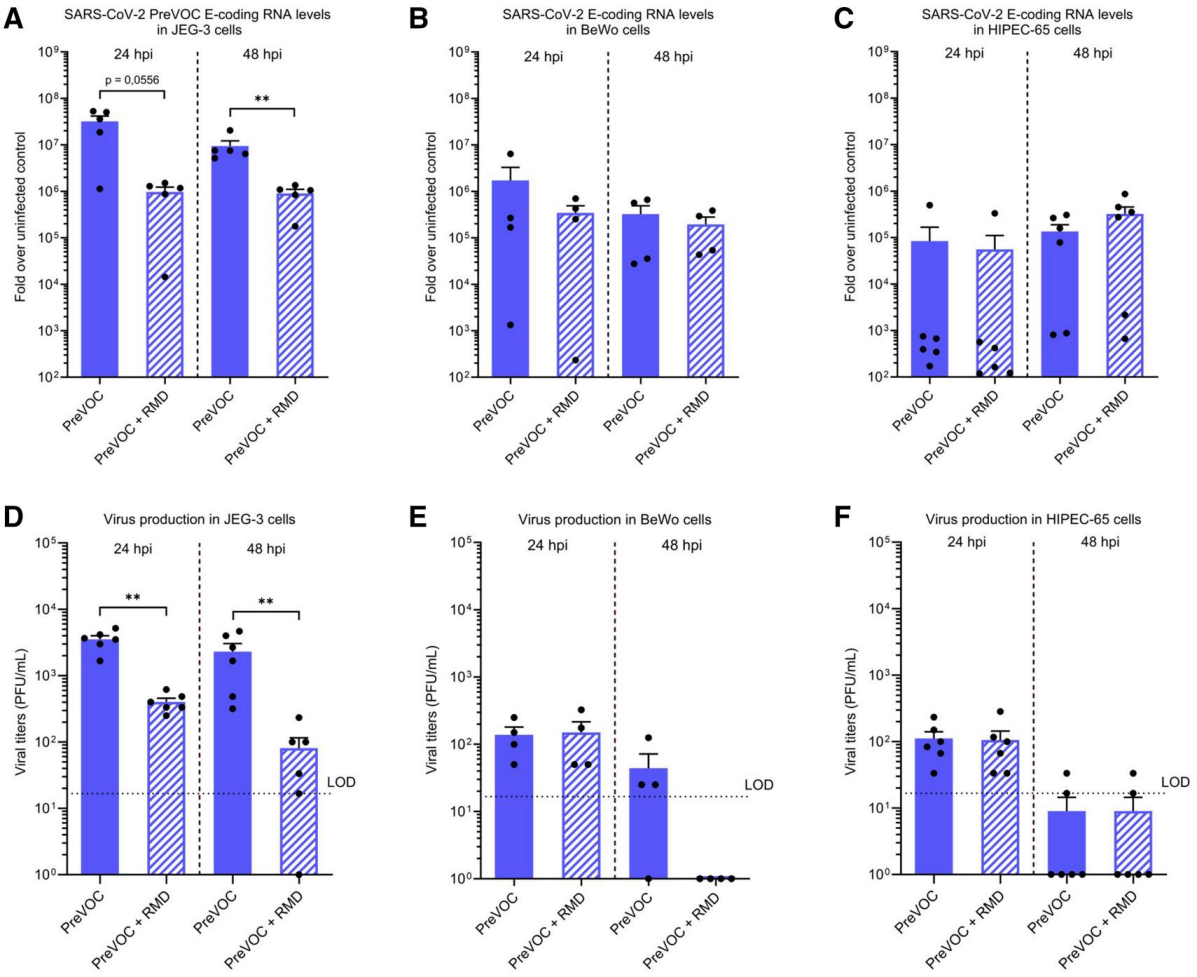
**JEG-3 cells are permissive to SARS-CoV-2 PreVOC strain compared to other trophoblastic cell lines**. Cells were infected at an MOI of 1 with the ancestral SARS-CoV-2 (PreVOC) strain and treatment with Remdesivir (RMD) was used as a positive control for viral replication. (**A**, **B**, **C**) Viral replication of SARS-CoV-2 PreVOC strain was evaluated by diagnostic RT-qPCR detecting E-coding RNA levels in JEG-3 (A; n = 6), BeWo (B; n = 4), and HIPEC-65 (C; n = 6) cells at 24 and 48 hpi. (**D**, **E**, **F**) Production of infectious viral particles in JEG-3 (D; n = 6), BeWo (E; n = 4), and HIPEC-65 (F; n = 6) cells at 24 and 48 hpi was determined using plaque assay. Data are shown as mean ± SEM (*P < 0.05, **P < 0.01; Mann–Whitney test). The black dashed line represents the limit of detection (LOD) of the plaque assay. hpi, hours post-infection; MOI, multiplicity of infection; PFU, Plaque-forming unit.

To evaluate the suitability of such alternative models for SARS-CoV-2 infection studies, we assessed the permissiveness of two cancerous trophoblastic cell lines (namely BeWo and JEG-3) and of the immortalized cell line HIPEC-65 to the PreVOC strain of SARS-CoV-2. Following exposure with SARS-CoV-2 PreVOC, supernatants and cells were collected at 24 and 48 hpi and analyzed as previously using RT-qPCR (Fig. 2A–C) and plaque assays (Fig. 2D–F). Of note, an internal quality control of efficient infection using Huh7.5 cells was systematically included in all experiments (data not shown). For all 3 cell lines, viral RNA was detected at every time point, but the levels of viral RNA were the highest in infected JEG-3 cells. They decreased significantly in the presence of RMD in JEG-3 cells at 24 and 48 hpi (Fig. 2A), whereas no difference was found with RMD treatment for the infected BeWo and HIPEC-65 cells (Fig. 2B and C). These results suggest that newly synthetized viral RNA was produced in JEG-3 cells but not in BeWo or in HIPEC-65 cells. In line with these observations, the plaque assays showed that in JEG-3 cells, the viral production was readily detected in a replication-dependent manner (Fig. 2D) and in the same order of magnitude as in infected primary vCTBs (Fig. 1E). On the other hand, in BeWo and HIPEC-65 cells at 24 and 48 hpi low (or sometimes undetectable) production of infectious particles was found (Fig. 2E and F), namely at the levels of the RMD background condition. Overall, these data provide evidence that the virus does not replicate efficiently in BeWo and HIPEC-65 cells. Hence, they should not be used to study infection by SARS-CoV-2. The JEG-3 cell line, on the other hand, seems to be an appropriate alternative model to study SARS-CoV-2 infection and to test antiviral drugs on trophoblastic cells *in vitro*.

### Viral entry factor expression differs among the trophoblastic cell lines

To better understand the differences in permissiveness among the trophoblastic cell lines, we analyzed several cellular features driving viral susceptibility, that is the capacity of the virus to enter a target cell. Trophoblastic cell lines have not been extensively characterized in that respect; thus, we assessed whether these cells express the factors reported as being required for SARS-CoV-2 entry in other cell types. The canonical entry pathway involving ACE2 and TMPRSS2 has been extensively described (Hoffmann *et al.*, 2020; Letko *et al.*, 2020; Walls *et al.*, 2020; Zhou *et al.*, 2020) but other entry routes mediated by alternative receptors, such as NRP1 (Cantuti-Castelvetri *et al.*, 2020; Daly *et al.*, 2020; Argueta *et al.*, 2022) and SRB1 (Wei *et al.*, 2020; Ramirez *et al.*, 2021) have also been reported. Moreover, after binding to a receptor, the virus can enter through the endosomal pathway and S is cleaved by CTSL instead of TMPRSS2 (Ou *et al.*, 2020; Koch *et al.*, 2021). With that in mind, we performed RT-qPCR to investigate the expression levels of these entry factors in the trophoblastic cell lines of Fig. 2 and in the human placental tissue as well as in Huh7.5 liver cells as a control.

**Figure 3.**
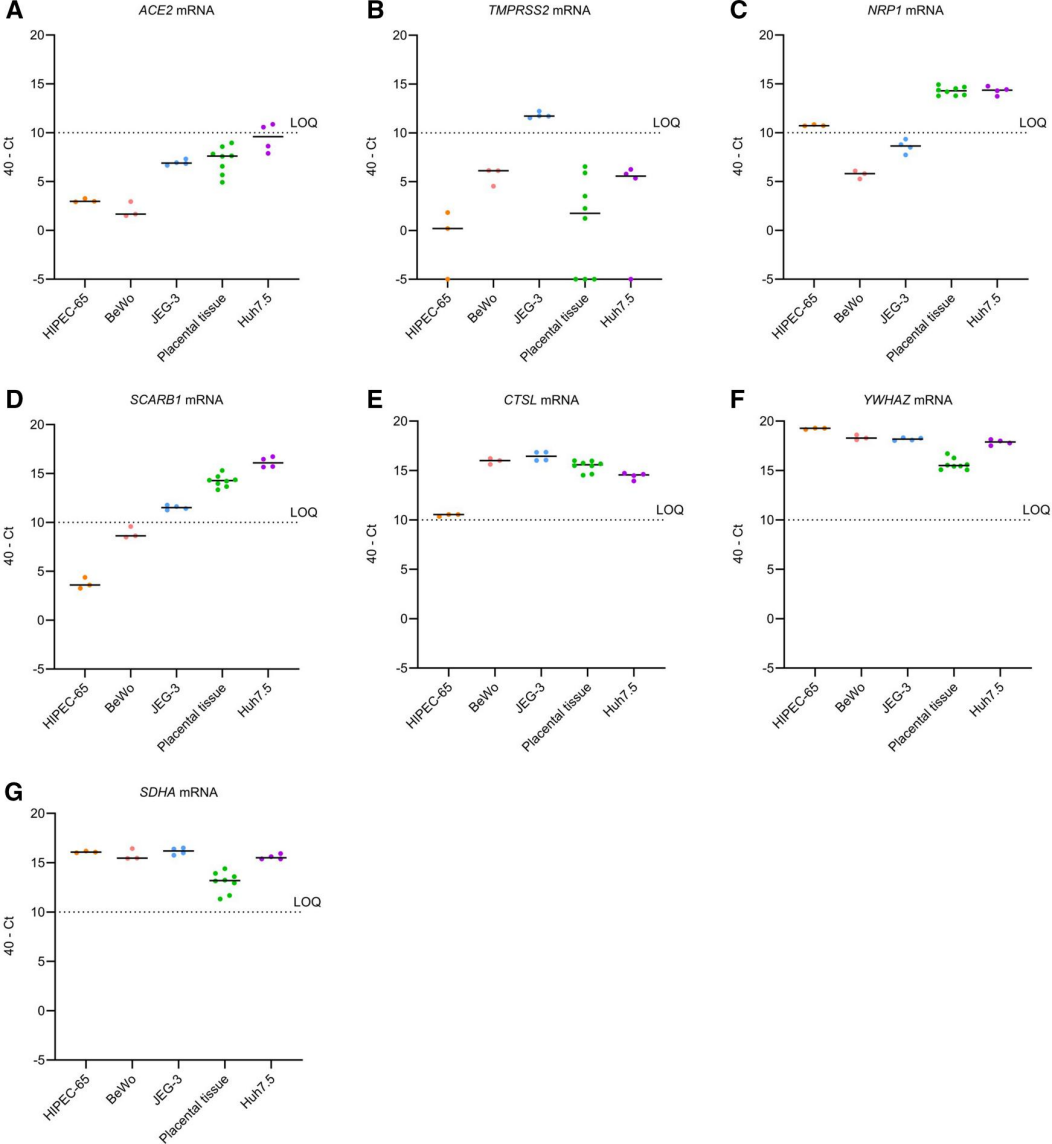
**Non-canonical SARS-CoV-2 entry factors are expressed in trophoblastic cell lines**. (**A**–**B**) Canonical (*ACE2* and *TMPRSS2*) and (**C**–**E**) non-canonical (*NRP1*, *SCARB1*, and *CTSL*) SARS-CoV-2 entry factor mRNA expression was determined by RT-qPCR in trophoblastic cell lines (HIPEC-65, n = 3; BeWo, n = 3; JEG-3, n = 4), placental tissue (n = 8), and the hepatoma cell line (Huh7.5, n = 4) used as a control. (**F**, **G**) Reference genes (*YWHAZ* and *SDHA*) mRNA expression was also measured by RT-qPCR, as a positive control for each cell types. Data are shown as the difference between the Cycle threshold (Ct) of the target gene and a Ct of 40, a value associated with total lack of expression. The black dashed line represents the limit of quantification (LOQ), that was set at a Ct of 30, a commonly accepted Ct value above which the expression of a gene is either barely detectable or absent.

Our results show that none of the cell types expressed reliably detectable amounts of mRNA coding for the canonical receptor ACE2, with all Ct over 30 (Fig. 3A) and only JEG-3 cells express mRNA encoding the canonical protease TMPRSS2, yet at low levels (Fig. 3B). *NRP1* and *SCARB1* mRNAs coding for alternative receptors were readily detected in placental tissue and Huh7.5 cells (Fig. 3C and D). *NRP1* mRNA was also expressed at very low level in HIPEC-65 cells while *SCARB1* mRNA was detected at low levels in JEG-3 cells (Fig. 3C and D). *CTSL* mRNA encoding the endosomal protease CTSL was highly expressed in most of the cell types tested (Fig. 3E). Moreover, the reference genes *YWHAZ* and *SDHA* were highly expressed in all cell types (Fig. 3F and G). These results suggest that HIPEC-65 and BeWo cells do not express the known required factors for viral entry and thus, that they are not susceptible to SARS-CoV-2, which is in line with the absence of significant detectable RNA replication and virus production in these cell lines (Fig. 2). JEG-3 cells express non-canonical receptor *SCARB1* mRNA and both canonical protease *TMPRSS2* mRNA and non-canonical protease *CTSL* mRNA, suggesting several possible entry pathways for the virus. Interestingly, Huh7.5 cells and human placental tissues express similar level of non-canonical entry factors.

**Figure 4.**
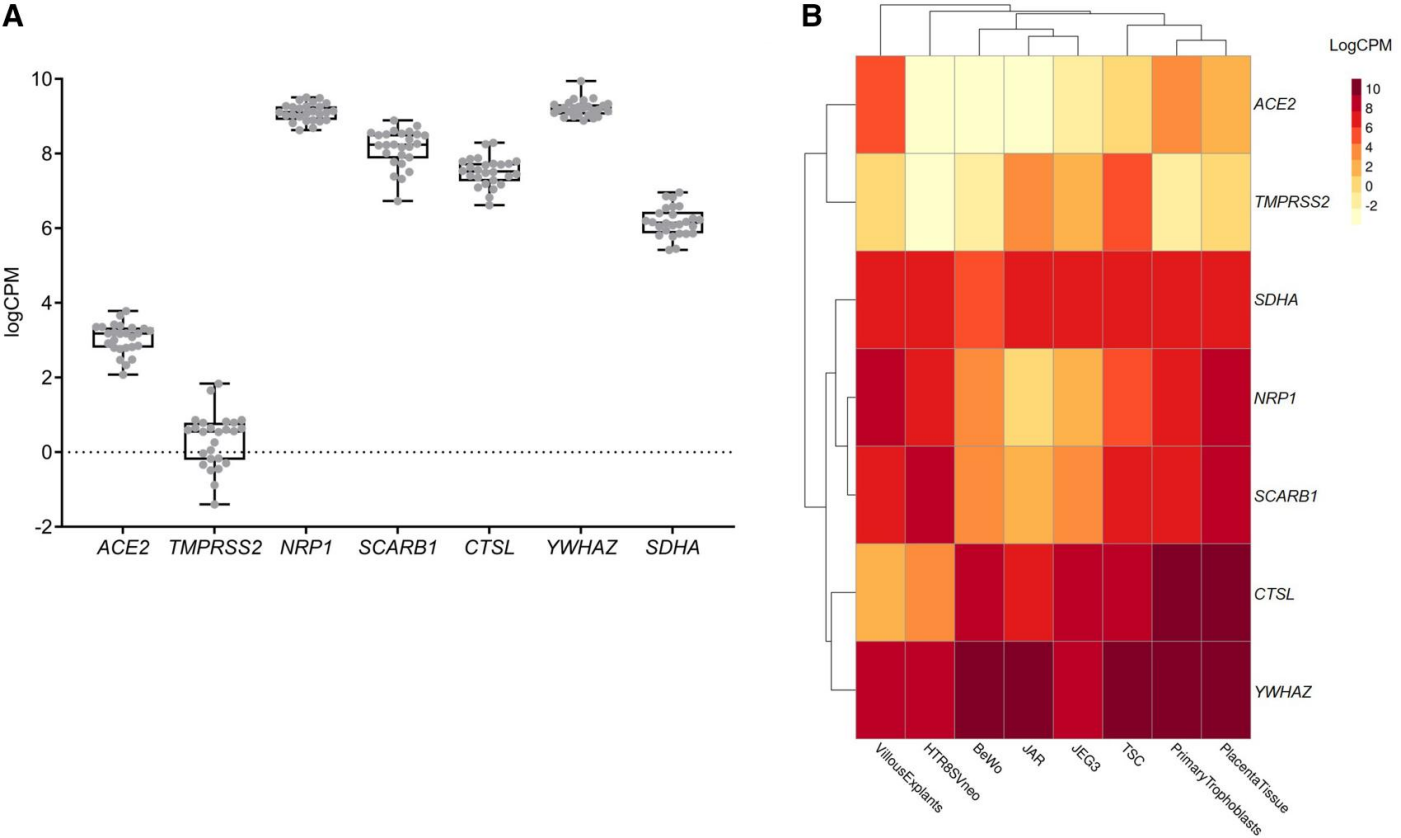
**SARS-CoV-2 entry factor expression differs depending on the trophoblastic model**. (**A**) Expression of key viral entry factors and reference genes was assessed by RNA sequencing of 26 placentas from uninfected donors. Data are represented as counts per million (CPM) and are shown as box and whisker plot. The box shows the median and the 25th and 75th percentiles, the whiskers indicate the lowest and the highest values and each gray dot represents a single donor. The black dashed line at logCPM = 0, represents the threshold of expression. (**B**) Heatmap of entry factors gene expression measured by RNA sequencing, between multiple trophoblastic models. Created with https://paquettelab.shinyapps.io/ComparativeTranscriptomicPlacentalModelAtlasApp/.

To further investigate the expression of these entry factors at the organ level, we performed mRNA sequencing (RNA-seq) on 26 term placenta biopsies collected from SARS-CoV-2 negative donors (Fig. 4A). The subsequent targeted mRNA expression analysis confirmed that the canonical entry factors *ACE2* and *TMPRSS2* are expressed at very low levels in term placental tissues, especially *TMPRSS2*, whose expression was markedly lower than that of alternative entry factor genes (*NRP1*, *SCARB1*, and *CTSL*) and reference housekeeping genes (*YWHAZ* and *SDHA)*. This pattern is in line with our RT-qPCR results, in which *ACE2* and *TMPRSS2* mRNA were below the limit of quantification in whole placental tissue (Fig. 3A and B). Although *ACE2* was not quantifiable by RT-qPCR, the RNA-seq data demonstrate that it is present at low level in term placental tissues. Notably, substantial inter-individual variability was observed across all entry factor genes, especially for *TMPRSS2* expression. This variability suggests that susceptibility of placental cells to SARS-CoV-2 may differ between individuals (Fig. 4A). To compare SARS-CoV-2 entry factor expression across multiple trophoblastic models (including primary trophoblasts) from independent datasets, we next took advantage of the CTPMA, recently developed and reported by Paquette’s team (Lapehn *et al.*, 2025). This tool integrates multiple independent RNA sequencing datasets to allow transcriptomic comparison between various placental models. The summary heatmap and the box plots for individual mRNAs generated using this resource (Fig. 4B and Supplementary Fig. S1) show that the gene expression patterns vary substantially depending on the trophoblastic model used (Fig. 4B). Placental tissue samples (n = 1083) exhibit profiles consistent with our RNA-seq data, characterized by low *ACE2* and *TMPRSS2* mRNA levels and higher expression of *NRP1*, *SCARB1*, *CTSL* as well as *YWHAZ* and *SDHA*. Interestingly, primary trophoblasts samples (n = 36) closely mirrored placental tissue, supporting the notion that whole placental RNA expression may be used as a proxy for primary trophoblast transcriptomic landscape in this context. The RNA sequencing data from BeWo and JEG-3 cells further aligned with our RT-qPCR data. They indeed show a lack of *ACE2* and *TMPRSS2* expression in BeWo cells, and only very low levels of *NRP1* and *SCARB1*, consistent with their limited permissiveness to SARS-CoV-2. In JEG-3 cells, low-level expression of *TMPRSS2* and *SCARB1* mRNAs was confirmed, and *ACE2* was undetectable. On the other hand, *NRP1*, undetectable by RT-qPCR, was expressed at low but measurable level in JEG-3 cells. *CTSL*, *YWHAZ*, and *SDHA* were highly expressed in both BeWo and JEG-3 cell, as we observed in RT-qPCR assays (Fig. 3). Overall, these findings show different patterns in SARS-CoV-2 entry factor expression across placental models. Among the cell lines examined, JEG-3 cells most closely resemble primary trophoblasts at the transcriptional level, supporting their relevance as a model system for studying SARS-CoV-2 entry into placental cells.

### SARS-CoV-2 enters trophoblastic cells via multiple pathways

**Figure 5.**
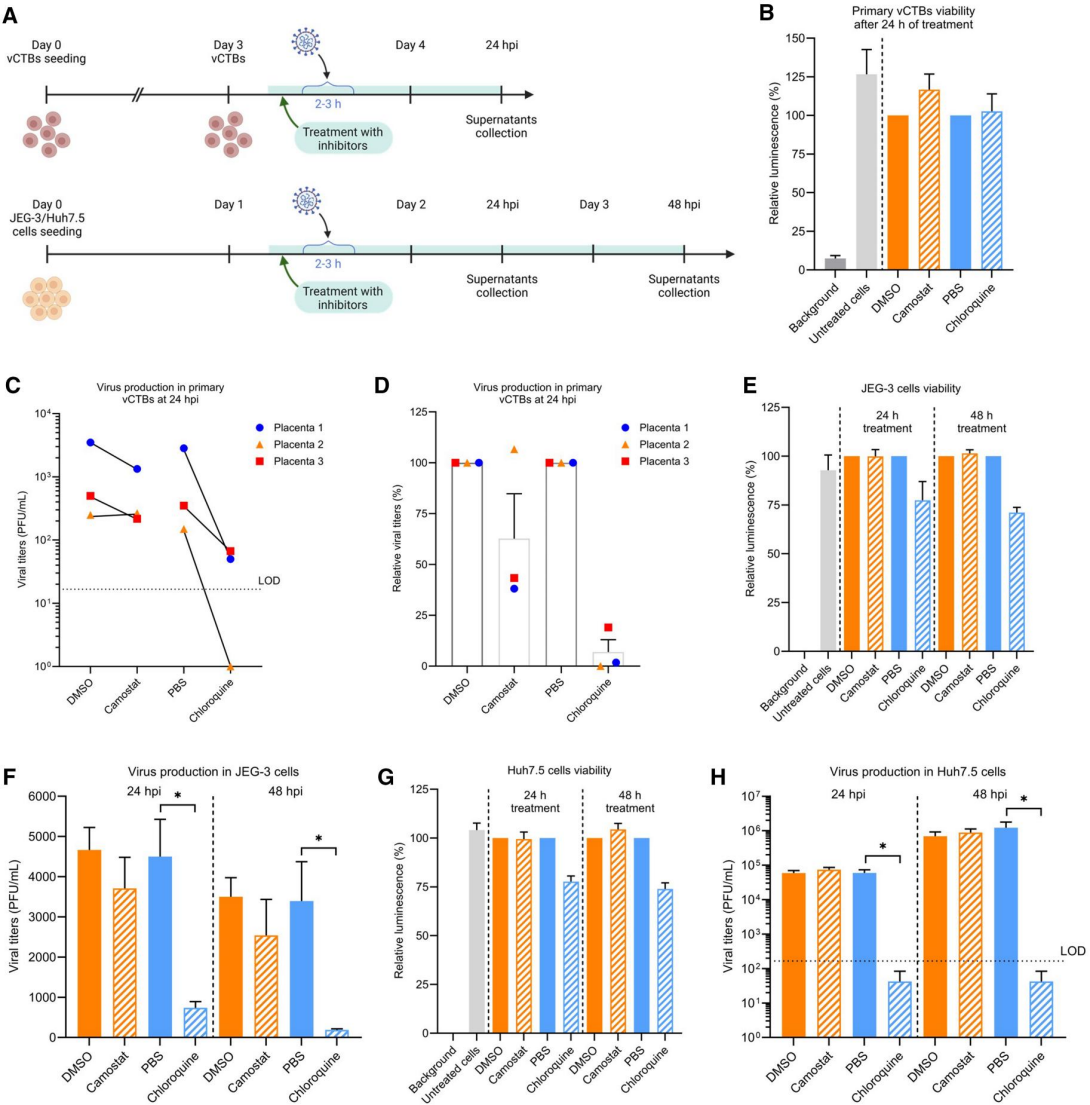
**SARS-CoV-2 PreVOC strain infects primary villous cytotrophoblasts, JEG-3 and Huh7.5 cells through the non-canonical entry pathway**. (**A**) Design of entry assay to evaluate the entry pathways used by PreVOC SARS-CoV-2. Created in BioRender. Vaillancourt, C. (2026) https://BioRender.com/oaa0jcq. (**B**, **E**, **G**) Cytotoxicity of the drugs was assessed by CellTiter-Glo and each treatment was compared to its appropriate vehicle control (0.1% DMSO or PBS). For primary vCTBs (**B**), data are shown as mean ± SEM (Mann–Whitney test). For JEG-3 (**E**) and Huh7.5 (**G**), experiment was performed with 6 technical replicates and data are shown as mean ± SD. (**C**, **D**) Viral replication was evaluated by plaque assay in primary vCTBs at 24 hpi, with each symbol representing a placenta from an individual donor (n = 3), (**F**) in JEG-3 cells (n = 4) and (**H**) in Huh7.5 cells (n = 4) at 24 and 48 hpi. Data are shown as mean ± SEM (* *P* < 0.05; Mann–Whitney test). The black dashed line represents the limit of detection (LOD) of the plaque assay. When no viral titer was detected, a value of 1 was arbitrarily assigned. hpi, hours post-infection; PFU, Plaque-forming unit; vCTBs, villous cytotrophoblasts.

To further investigate the entry pathway(s) used by SARS-CoV-2, vCTBs, JEG-3, and Huh7.5 cells were pre-treated with 20 µM Camostat mesylate, a serine protease inhibitor targeting TMPRSS2 (Mantzourani *et al.*, 2022) or 20 µM chloroquine phosphate, an antimalarial drug that prevents the activation of endosomal proteases such as CTSL through increasing endosomal pH (Savarino *et al.*, 2003; Keyaerts *et al.*, 2004), before infection by the PreVOC SARS-CoV-2 strain (Fig. 5A). As reference controls, cells were treated with the respective drug vehicle (PBS or DMSO). Virus-containing cell supernatants were collected at 24 hpi for the primary trophoblasts and at 24 hpi and 48 hpi for Huh7.5 and JEG-3 cells and used for plaque assays (Fig. 5A). The cytotoxicity of the drugs was assessed by evaluating cell viability with CellTiter-Glo assays. Primary vCTBs did not display any significant decrease in cell viability upon Camostat mesylate or chloroquine phosphate treatment in comparison to their respective vehicle-treated control (Fig. 5B). The secretion of infectious particles was reduced by chloroquine treatment of the primary cells from all three donor placentas (Fig. 5C and D). This viral production was abolished in cells from Placenta No. 2 upon chloroquine phosphate treatment and there was no effect of Camostat mesylate treatment, suggesting that the virus enters the cells exclusively through the non-canonical endosomal (TMPRSS2-independent) pathway. Interestingly, with cells from Placentas No. 1 and No. 3, the viral production was decreased by around 96% with chloroquine phosphate treatment but not completely lost, whereas it was decreased by around 61% with Camostat mesylate treatment (Fig. 5C and D). This supports that when the endosomal pathway is inhibited, the virus is still able to enter the cell through the TMPRSS2-dependent canonical pathway and to resume a full life cycle. These results strongly suggest that the virus mainly uses the non-canonical entry pathway to infect primary vCTBs *in vitro* but may alternatively use the canonical one, most likely depending on the availability of entry factors.

In JEG-3 cells, chloroquine phosphate treatment induced only a moderate decrease in viability (Fig. 5E) and a decrease in virus production of around 83% at 24 hpi and 94% at 48 hpi compared to the vehicle control (Fig. 5F), demonstrating a strong dependency of the viral entry on the endosomal pathway. In contrast, Camostat mesylate treatment induced a slight decrease of around 20% of viral production at 24 hpi and around 27% at 48 hpi (Fig. 5F), suggesting that the canonical TMPRSS2-related pathway can be used to a lesser extent. These results are in line with mRNA expression analysis showing that TMPRSS2 is expressed at low levels while the endosomal protease CTSL is highly expressed (Figs 3B and E and 4B). According to these results, the PreVOC SARS-CoV-2 strain appears to infect JEG-3 cells via the same pathway as that in primary trophoblasts *in vitro*, namely primarily through the non-canonical endosomal pathway.

As expected from a previous report (Dittmar *et al.*, 2021), treatment of Huh7.5 cells with Camostat mesylate had no impact on the viral production, whereas treatment with chloroquine phosphate abolished it at 24 and 48 hpi (a decrease of more than 99%) (Fig. 5G and H). These results suggest that the PreVOC strain can infect Huh7.5 cells only through the non-canonical endosomal pathway.

### SARS-CoV-2 variants differentially infect human primary trophoblast cells

All the experiments described above were done testing the ancestral PreVOC SARS-CoV2, the strain that spread through the world and caused the COVID-19 pandemic in 2020. But since the first dissemination of the virus across the planet, the virus has evolved, and new variants arose and became dominant. At the protein sequence level, these variants differed from the ancestral strain primarily in the Spike protein, which plays a pivotal role in the entry of the virus into the cell (Flores-Vega *et al.*, 2022). Therefore, we hypothesized that the permissiveness of the trophoblasts might change depending on the infecting SARS-CoV-2 variant. To assess this, we infected JEG-3 cells and vCTBs isolated from two donor placentas with variants of concern Alpha (isolate B.1.1.7), Beta (isolate B.1.351), and Delta (isolate B.1.617.2).

**Figure 6.**
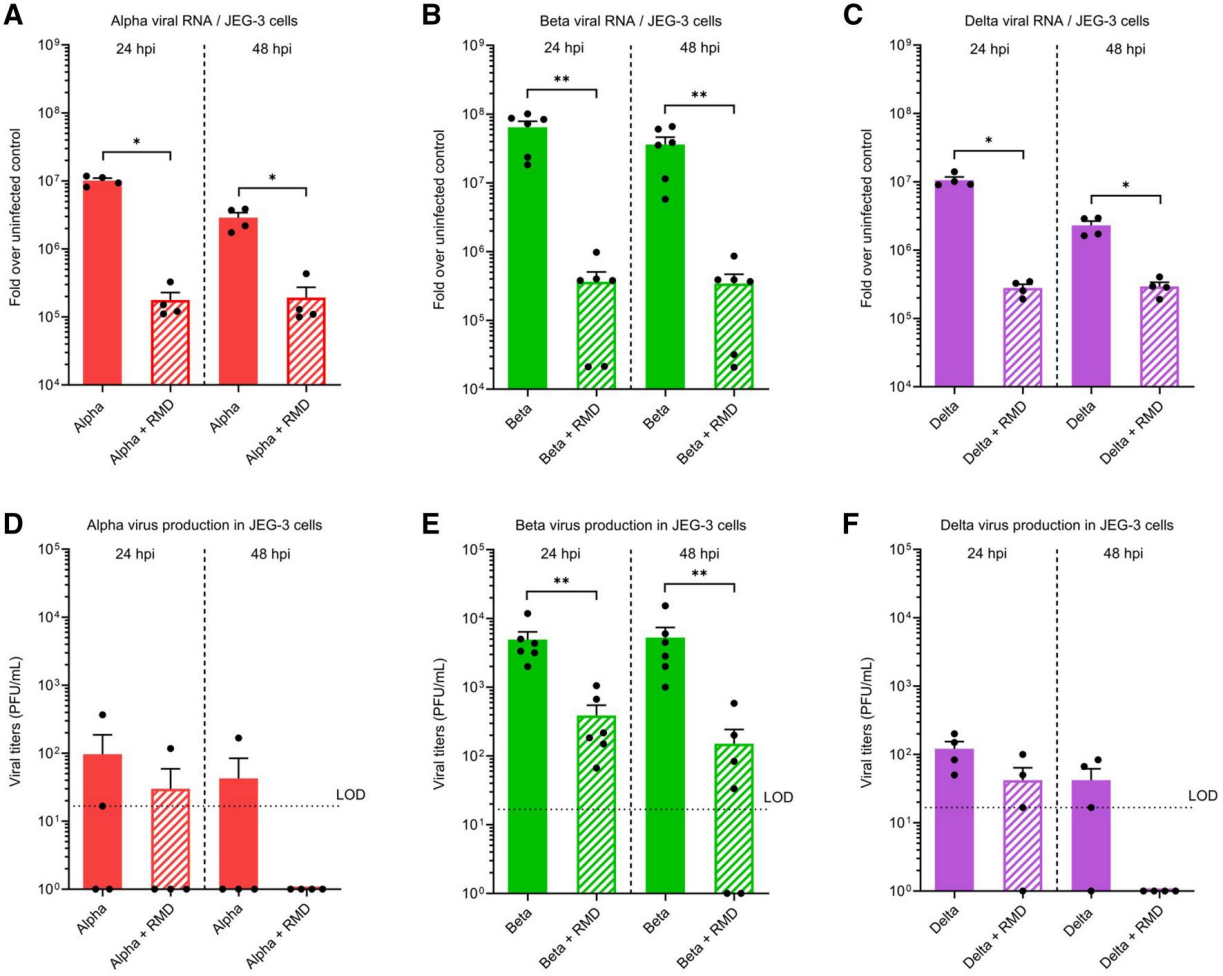
**SARS-CoV-2 variant Beta is more effective at infecting JEG-3 cells**. JEG-3 cells were infected with SARS-CoV-2 Alpha (B.1.1.7) (n = 4), Beta (B.1.351) (n = 6), or Delta (B.1.617.2) (n = 4) strains at an MOI of 1. Remdesivir (RMD) treatment of the infected cells was used as a control for viral replication. (**A**, **B**, **C**) Viral replication of the different strains of SARS-CoV-2 was evaluated by diagnostic RT-qPCR detecting E-coding RNA levels. (**D**, **E**, **F**) Concentration of infectious viral titers (PFU/ml) in the supernatants of infected JEG-3 cells was evaluated by plaque assay. Data are shown as mean ± SEM (*P < 0.05, **P < 0.01; Mann–Whitney test). The black dashed line represents the limit of detection (LOD) of the plaque assay. When no viral titer was detected, a value of 1 was arbitrarily assigned. hpi, hours post-infection; MOI, multiplicity of infection; PFU: plaque-forming unit; vCTBs, villous cytotrophoblasts.

RT-qPCR and plaque assays detected replication of the three VOCs tested in JEG-3 cells since RMD treatment significantly decreased the amount of viral RNA and extracellular infectious particles produced (Fig. 6A–F). In addition, the comparison of viral RNA intracellular abundance between the different variants and the PreVOC strain (Fig. 2A) showed a slightly decreased replication of variants Alpha and Delta but a higher replication of variant Beta (Fig. 6A–C). Plaque assays showed very low production of infectious particles following infection with Alpha and Delta variants which was close to the limit of detection and of specificity since no differences were observed upon RMD treatment at 24 hpi (Fig. 6D and F). In contrast, consistent with the RT-qPCR data, the particle production of the variant Beta was comparable to that of the PreVOC strain (Figs 2D and 6E ). These data suggest that JEG-3 cells display a higher permissiveness to the Beta variant than to the Alpha and Delta variants.

For vCTBs, our data from RT-qPCR and plaque assays on a limited number of donor placentas (n = 2) showed differences in the amount of viral RNA and infectious particles produced by the primary cells depending on the infecting variant (Supplementary Fig. S2A and B). Viral E-coding RNA was detected for each of the VOCs in the vCTBs with the highest levels found for PreVOC and Delta. The plaque assay results followed the same pattern with PreVOC-infected cells producing more viral particles than Delta-infected cells, whereas cells infected with Alpha or Beta VOCs appeared to produce little amount of virus, if any (Supplementary Fig. S2B). Interestingly, the immunostaining of the viral protein N showed a strong signal in PreVOC-, Delta-, and Beta-infected cells but in Alpha-infected cells a weaker signal was observed (Supplementary Fig. S2C). Based on the analysis of cells from two donor placentas, these results raise the hypothesis that primary vCTBs are more permissive to the PreVOC strain than to any other variant tested and that there are differences in viral fitness depending on the variant. This contrasts with JEG-3 cells in which the variant Beta replicated as well as the PreVOC strain (Figs 2 and 6). Further experiments on vCTBs from additional donors are required to validate the observed differences between primary and immortalized trophoblasts in their SARS-CoV-2 permissiveness and correlate the phenotypes with the expression of the viral entry factors.

## Discussion

We showed that SARS-CoV-2 PreVOC strain completes a full-replication cycle in primary vCTBs, notably resulting in producing new infectious viral particles. This is in accordance with reports showing CTBs permissiveness to SARS-CoV-2 (Lu-Culligan *et al.*, 2021; Argueta *et al.*, 2022). Other studies using early-pregnancy models have shown that infection can impair vCTBs differentiation into STBs (Chen *et al.*, 2023), suggesting that infection of vCTBs *in vivo* may disrupt both placental structure and STB-mediated functions.

The STB is the first layer of the placenta directly in contact with the maternal blood and thus represents the frontline target of the virus. Its infection could enable viral access to underlying CTBs, although STB may also act as a barrier that restricts viral dissemination. In this study, we used primary vCTBs isolated from healthy term placentas–a model known to spontaneously fuse over time (Kliman *et al.*, 1986; Lanoix *et al.*, 2008) – allowing investigation of both vCTBs and STB-like syncytia. However, due to insufficient cell density, spontaneous fusion did not occur under our conditions, preventing direct comparison of CTB and STB infection rates. Future studies using higher-density vCTB cultures may clarify whether SARS-CoV-2 affects syncytialization at term.

Depending on the timing of infection throughout pregnancy, such alterations might hinder the proper development of the fetus and maternal health. Since our model recapitulates the vCTBs at term, it would be interesting to adapt our experimental design to use a higher cell density and infect the vCTBs immediately after seeding. Measuring the formation of syncytia over the course of 72 h of culture would allow to evaluate whether the virus hinders the syncytialization process of vCTBs in late pregnancy.

The comparison of our results with others' reported trophoblastic models illustrates the variability in replication kinetics. Organotypic cultures of precision-cut placenta slices (late pregnancy model) sustained viral production up to 120 hpi, whereas trophoblast stem cell-derived EVTs and STBs (early pregnancy model) displayed a peak of replication at 24 hpi, similar to our observation in vCTBs (Fahmi *et al.*, 2021; Kallol *et al.*, 2023). These discrepancies could reflect differences in the trophoblastic models used and in the experimental designs. In this study, vCTBs were kept in culture for a 7-day period, during which trophoblast viability is known to decline after 72 h (Kliman *et al.*, 1986; Lanoix *et al.*, 2008). Another possibility accounting for these differences could be the inter-donor variability in permissiveness of trophoblasts isolated from different placentas. Fahmi *et al.* (2021) reported that only 2 of 7 placental donors produced highly infectious titers in precision-cut slices and suggested that susceptibility correlated with ACE2 expression. Although we could not measure entry-factor expression directly in the infected vCTBs, our RNA-seq analysis of placental biopsies and primary trophoblasts showed donor-dependent variation in key entry factors, particularly TMPRSS2, a critical mediator of the canonical entry pathway. The similar range of expression observed in placental tissue and isolated trophoblasts further suggests that whole-placenta RNA provides a reasonable approximation of vCTB entry-factor expression in this context.

We have also tested the infection efficiency of the PreVOC SARS-CoV-2 strain on 3 different trophoblastic cell lines, namely BeWo, JEG-3, and HIPEC-65. Among these models, BeWo and JEG-3 cells are probably the most widely used in the field of placental research. Both are derived from a brain metastasis of a choriocarcinoma and during their adaptation to cell culture, they maintained distinct trophoblastic properties (Hertz, 1959; Pattillo and Gey, 1968; Kohler and Bridson, 1971). BeWo cells exhibit a term vCTB-like phenotype and fuse to form syncytia upon forskolin (FK) stimulation, achieving full morphological but biochemically incomplete differentiation (Wice *et al.*, 1990; Orendi *et al.*, 2010). On the other hand, JEG-3 cells are non-fusogenic, vCTB-like cells that can differentiate biochemically upon FK treatment but not morphologically (Borges *et al.*, 2003; Al-Nasiry *et al.*, 2006; Vargas *et al.*, 2008). JEG-3 cells express HLA-G, a marker of invasive EVTs, and thus, are commonly used as a model of EVT-like trophoblast cells (Pastuschek *et al.*, 2021). Less commonly used, HIPEC-65 cells are proliferative and highly invasive EVT-like cells. They were immortalized from first-trimester EVTs with simian virus 40 (SV40) large T antigen and they express EVT markers (Pavan *et al.*, 2003). Our results show that neither BeWo cells nor HIPEC-65 cells are permissive to SARS-CoV-2 infection. While this could be interpreted as evidence against permissiveness of CTBs at term and EVTs in early pregnancy, our data on primary CTBs and a prior study on permissiveness during early pregnancy (Kallol *et al.*, 2023), indicate instead that both trophoblastic cell types are permissive to SARS-CoV-2 and that these two cell lines are not suitable models for studying SARS-CoV-2 infection. This conclusion is strengthened by the very low or absent expression of both canonical (ACE2, TMPRSS2) and non-canonical (SRB1 and NRP1) viral entry factors in BeWo and HIPEC-65 cells. The present study is the first to investigate HIPEC-65 cells potential permissiveness to SARS-CoV-2 while previous studies reported similar results regarding lack of effective infection in the BeWo cell line (Lu-Culligan *et al.*, 2021; Tallarek *et al.*, 2021).

Most importantly, our data highlight that JEG-3 cells allow replication of SARS-CoV-2 to levels similar to those in the primary vCTBs *in vitro* and therefore, represent a suitable model to study trophoblastic infection. These results contrast with the published data from Tallarek and colleagues showing the absence of production of infectious particles by JEG-3 cells following exposure to SARS-CoV-2. However, this study lacked appropriate infection controls, and the observed differences between our studies might result from the use of different viral strains or cell passages (Tallarek *et al.*, 2021). Our conclusion about effective viral replication is strongly supported by the fact that treatment with RMD, a potent antiviral drug, robustly reduced the intracellular levels of viral RNA and extracellular infectious particles. Those results were confirmed by treatment with NIR, another inhibitor of viral replication (data not shown).

Interestingly, entry assays show that SARS-CoV-2 PreVOC strain enters the vCTBs through non-exclusive canonical and non-canonical pathways. In two placentas, the inhibition of either TMPRSS2 or the endosomal pathway resulted in a decrease of viral production without its complete disruption. It should be noted that the impairment of the endosomal pathway had the greatest impact on the viral production. In addition, cells from Placenta No. 2 were not able to produce any viral particles following endosomal pathway inhibition, and TMPRSS2 inhibition had no impact on the viral production (Fig. 5C and D). These data suggest that the endosomal pathway is the main entry route of SARS-CoV-2 in trophoblast cells. However, endosomal inhibition did not fully suppress infection in all donors, suggesting that multiple entry routes may be used and/or that inter-individual differences in entry factor expression (especially for TMPRSS2) likely influence pathway preference and overall permissiveness. Such variability may help explain conflicting reports about trophoblast susceptibility in the literature. It will be relevant to further explore how the virus uses either entry pathway depending on the factors available. Interestingly, our data with JEG-3 cells revealed a similar entry profile. Endosomal pathway inhibition resulted in the highest decrease in viral production while inhibition of TMPRSS2 had minimal effect (Fig. 5F). This behavior aligns with their expression of key entry factors: high levels of SRB1 and CTSL, and low but detectable TMPRSS2. Consistent with prior reports, ACE2 and TMPRSS2 expression at the materno–fetal interface is low and decreases with gestational age (Pringle *et al.*, 2011; Li *et al.*, 2020; Bloise *et al.*, 2021; Ouyang *et al.*, 2021). Our RNA-seq analyses likewise revealed high expression of alternative factors (*NRP1*, *SCARB1*, *CTSL*) and very low *ACE2*/*TMPRSS2* levels in both primary trophoblasts and placental tissue, supporting a predominant reliance on endosomal entry in these models.

To fully characterize SARS-CoV-2 entry mechanisms in trophoblasts, further studies are needed to test simultaneous inhibition of canonical and endosomal pathways and to assess whether additional routes such as the recently described MMP-2/9-dependent entry pathway (Benlarbi *et al.*, 2022), contribute to infection. As JEG-3 cells secrete both MMP-2 and MMP-9 (Karmakar *et al.*, 2004; Huang *et al.*, 2017; Clabault *et al.*, 2018), it is tempting to speculate that virus entry into trophoblasts involves these metalloproteinases. Moreover, to precisely identify the receptor(s) involved in this entry, further experiments are required, such as assessing replication upon inhibition of NRP1 and SRB1 receptors with blocking antibodies (Argueta *et al.*, 2022). Overall, our data bring strong evidence that JEG-3 cells are a proper model to study SARS-CoV-2 infection of trophoblastic cells.

Most experiments in this study were performed with the ancestral strain (PreVOC) that spread across the world in early 2020. This strain, which had already diverged from the original Wuhan isolate notably with the mutation D614G in the Spike protein (Korber *et al.*, 2020), was later replaced by newly emerging variants carrying mutations that increased transmissibility or immune escape. Among these, Alpha (B.1.1.7), Beta (B.1.351), and Delta (B.1.617.2) were classified as VOCs by the World Health Organization, and became sequentially dominant from December 2020 to October 2021 (Dubey *et al.*, 2021). Of note, North American epidemiological studies revealed that risks for adverse maternal and fetal outcomes were at their highest during the Delta wave period (July 2021–September 2021) (DeSisto *et al.*, 2021; McClymont *et al.*, 2022; Carlson *et al.*, 2024), with a rate of stillbirth rising to 2.70% of COVID-19 deliveries compared to 0.63% of deliveries without COVID-19 in the USA (DeSisto *et al.*, 2021). Then, the Omicron lineage took over for a year followed by the emergence of Omicron-derived sub-lineages (Wang *et al.*, 2023; Holmes, 2024). This evolution of the virus as well as increased immunization (through vaccination or previous infection) among the population was associated with decreased risks for adverse outcomes, albeit still higher than in non-infected pregnant persons (McClymont *et al.*, 2022; Carlson *et al.*, 2024). To explore whether viral evolution affected trophoblastic permissiveness, we tested the susceptibility of JEG-3 cells and primary vCTBs to the VOCs available in our laboratory, namely Alpha, Beta, and Delta variants. JEG-3 cells were infected by all three VOCs, but the infectious titers varied depending on the strain. Interestingly, infection with Alpha and Delta variants produced low viral yields, suggesting altered requirement for assembly and/or egress compared to PreVOC and Beta, whereas infection with the Beta variant resulted in infectious titers comparable to PreVOC. In contrast, we observed a reduced permissiveness to all three VOCs relative to PreVOC from primary vCTBs isolated from two donors. These results on a limited number of donors strongly support that the observed differences between primary and immortalized trophoblasts in their SARS-CoV-2 permissiveness may be due to interindividual variability in the expression of the viral entry factors. Although these results require confirmation in a larger cohort, they suggest that virus evolution may have decreased SARS-CoV-2 trophoblastic tropism, potentially through changes in the entry route used by the virus. Based on the epidemiological data available for the Delta wave period, it was surprising that Delta was not as efficient as PreVOC in infecting the trophoblasts. The adverse pregnancy outcomes observed during its high prevalence period could then result from indirect effects of the maternal systemic infection rather than from placenta infection *per se*. However, further investigation is required to fully characterize the permissiveness of vCTBs to the previously mentioned VOCs and more recent variants.

In conclusion, we confirmed the permissiveness of primary term vCTBs to SARS-CoV-2 PreVOC *in vitro* and highlighted that the virus could use alternatively several entry routes depending on the entry factors availability. Our data further demonstrate that JEG-3 cells could be used as a cost-efficient and convenient model to further investigate altered cellular pathways upon SARS-CoV-2 infection.

## Supplementary Material

gaag015_Supplementary_Data

## Data Availability

The data supporting the findings of this study are available from the corresponding authors, C.V. and L.C.-C. The raw RNA sequencing data are openly available in Gene Expression Omnibus (GEO) with the accession number GSE318446.
