# Supplementary material for: SARS-CoV-2 infects human primary cytotrophoblasts mainly through a non-canonical entry route
Source: Mol Hum Reprod. 2026 Feb 26;32(1):gaag015. doi: 10.1093/molehr/gaag015 (PMC13019026; doi:10.1093/molehr/gaag015)
Supplement: gaag015_Supplementary_Data [file gaag015_supplementary_data.pdf]

Supplementary Information

SARS-CoV-2 infects human primary cytotrophoblasts mainly through a non-canonical entry route

Hélène Pinatel, Marie-Ève Brien, Mathilde Broquière, Marie-Pier Scott-Boyer, Arnaud Droit, Sylvie Girard, Géraldine Delbès, Laurent Chatel-Chaix, Cathy Vaillancourt

Supplementary Figure S1: Inter-individual variability among donors is illustrated by different degree of expression of viral entry factors.

Supplementary Figure S2: Human primary villous trophoblasts seem less permissive to SARS-CoV-2 variants Alpha, Beta and Delta than to PreVOC strain.

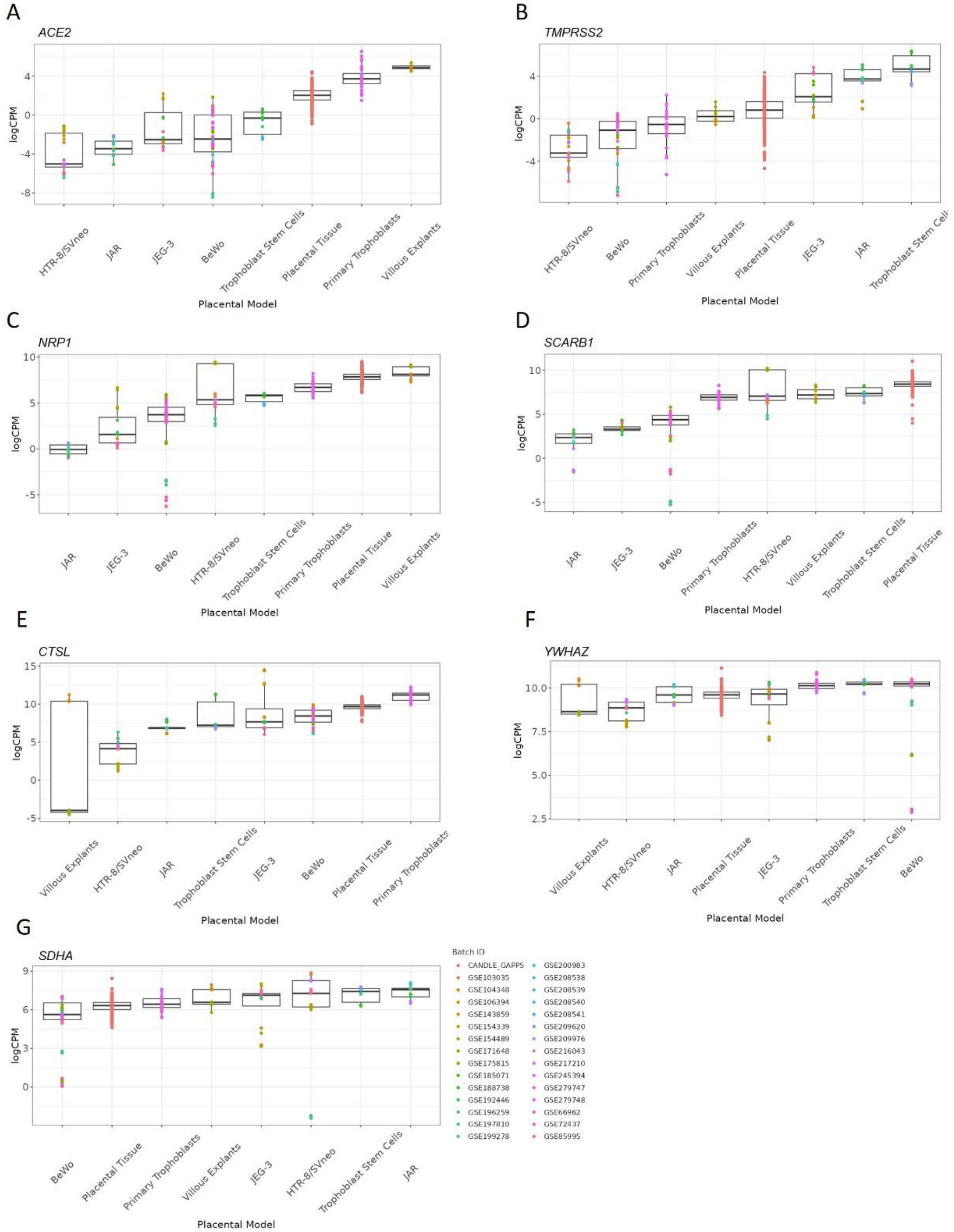

Supplementary Figure S1: Inter-individual variability among donors is illustrated by different degree of expression of viral entry factors

RNA expression of *ACE2* (**A**), *TMPRSS2* (**B**), *NRP1* (**C**), *SCARB1* (**D**), *CTSL* (**E**), *YWHAZ* (**F**) and *SDHA* (**G**) was assessed by RNA sequencing and they were compiled to allow comparison between placental models by Alison Paquette's team (Lapehn et al., 2025). Data are shown as box plots with the median. Each dot represents an individual donor or biological replicate. Each color refers to the dataset from which the data were extracted, identified by its GSE

number. Created with https://paquettelab.shinyapps.io/ComparativeTranscriptomicPlacentalModelAtlasApp/. CPM: counts per million.

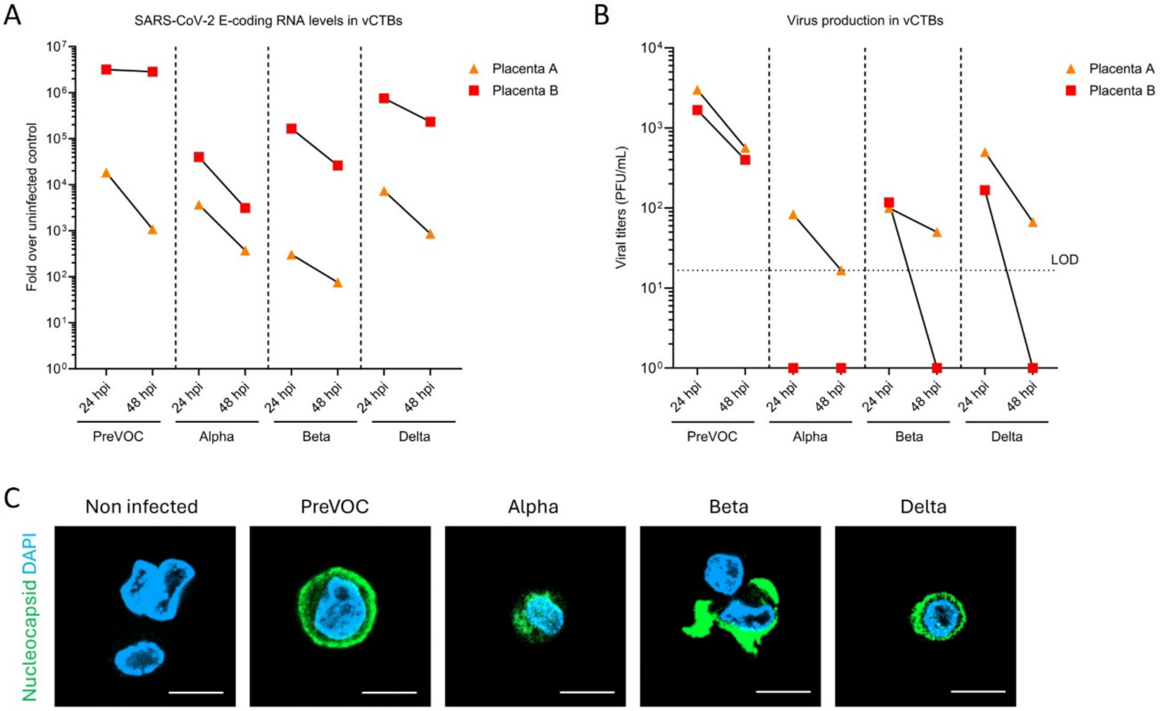

Supplementary Figure S2: Human primary villous trophoblasts seem less permissive to SARS-CoV-2 variants Alpha, Beta and Delta than to PreVOC strain.

Primary vCTBs cells were infected with SARS-CoV-2 PreVOC strain, Alpha (B.1.1.7), Beta (B.1.351) or Delta (B.1.617.2) at a MOI of 1. (**A**) Viral replication of the different strains of SARS-CoV-2 was evaluated by diagnostic RT-qPCR detecting E-coding RNA levels. (**B**) Infectious viral titers in the supernatants of infected primary vCTBs were assessed by plaque assay. Each symbol represents a placenta from an individual donor (n = 2). The black dashed line represents the limit of detection (LOD) of the plaque assay. When no viral titer was detected, a value of 1 was arbitrarily assigned. (**C**) vCTBs infected with various SARS-CoV-2 strain were detected by immunostaining of the Nucleocapsid SARS-CoV-2 protein in green and cell nuclei were counterstained with DAPI (blue). Cells were observed with confocal microscopy at 63x objective. Scale bar 10 µm. hpi: hours post-infection; MOI: multiplicity of infection; vCTBs: villous cytotrophoblasts.
